# Comprehensive Landscape of Immune Infiltration and Aberrant Pathway Activation in Ischemic Stroke

**DOI:** 10.3389/fimmu.2021.766724

**Published:** 2022-01-24

**Authors:** Rongrong Liu, Pingping Song, Xunhu Gu, Weidong Liang, Wei Sun, Qian Hua, Yusheng Zhang, Zhengang Qiu

**Affiliations:** ^1^ Department of Neurology and Stroke Center, The First Affiliated Hospital, Jinan University, Guangzhou, China; ^2^ Department of Oncology, The First Affiliated Hospital of Gannan Medical University, Ganzhou, China; ^3^ Department of Neurology, Ganzhou People’s Hospital, Ganzhou, China; ^4^ Department of Neurology, The Second Affiliated Hospital of Nanchang University, Nanchang, China; ^5^ Clinical Neuroscience Institute, Jinan University, Guangzhou, China; ^6^ Department of Anesthesiology, The First Affiliated Hospital of Gannan Medical University, Ganzhou, China

**Keywords:** ischemic stroke, immune infiltration, immune microenvironment, pathway, inflammation

## Abstract

Ischemic stroke (IS) is a multifactorial disease caused by the interaction of multiple environmental and genetic risk factors, and it is the most common cause of disability. The immune microenvironment and inflammatory response participate in the whole process of IS occurrence and development. Therefore, the rational use of relevant markers or characteristic pathways in the immune microenvironment will become one of the important therapeutic strategies for the treatment of IS. We collected peripheral blood samples from 10 patients diagnosed with IS at the First Affiliated Hospital of Gannan Medical University and First Affiliated Hospital, Jinan" University, and from 10 normal people. The GSE16561 dataset was downloaded from the Gene Expression Omnibus (GEO) database. xCell, gene set enrichment analysis (GSEA), single-sample GSEA (ssGSEA) and immune-related gene analysis were used to evaluate the differences in the immune microenvironment and characteristic pathways between the IS and control groups of the two datasets. xCell analysis showed that the IS-24h group had significantly reduced central memory CD8+ T cell, effector memory CD8+ T cell, B cell and Th1 cell scores and significantly increased M1 macrophage and macrophage scores. GSEA showed that the IS-24h group had significantly increased inflammation-related pathway activity(myeloid leukocyte activation, positive regulation of tumor necrosis factor biosynthetic process, myeloid leukocyte migration and leukocyte chemotaxis), platelet-related pathway activity(platelet activation, signaling and aggregation; protein polymerization; platelet degranulation; cell-cell contact zone) and pathology-related pathway activity (ERBB signaling pathway, positive regulation of ERK1 and ERK2 cascade, vascular endothelial growth factor receptor signaling pathway, and regulation of MAP kinase activity). Immune-related signature analysis showed that the macrophage signature, antigen presentation-related signature, cytotoxicity-related signature, B cell-related signature and inflammation-related signature were significantly lower in the IS-24h group than in the control group. In this study, we found that there were significant differences in the immune microenvironment between the peripheral blood of IS patients and control patients, as shown by the IS group having significantly reduced CD8+ Tcm, CD8+ Tem, B cell and Th1 cell scores and significantly increased macrophage and M1 macrophage scores. Additionally, inflammation-related, pathological, and platelet-related pathway activities were significantly higher in the IS group than in the control group.

## Introduction

Ischemic stroke (IS) is a multifactorial disease caused by the interaction of multiple environmental and genetic risk factors. It is the most common cause of disability and creates a heavy burden on society and families (1). The current effective drug treatment for IS intravenous thrombolysis [tissue plasminogen activator (t-PA)], but because of its narrow therapeutic window and the possibility of severe bleeding, the scope of benefit for patients is small (2, 3). However, the immune microenvironment and inflammatory response are involved in the whole process of IS occurrence and development and are closely related to the severity and prognosis of IS (4). Therefore, exploring the immune microenvironment and inflammatory mechanism of IS through the reasonable use of relevant markers or characteristic inflammatory pathways in the immune microenvironment to reduce neuronal damage will become one of the important therapeutic approaches for the treatment of IS.

Recently, the inflammatory immune response has been shown to participate in all stages of the pathological progression of IS, including the production of inflammatory mediators and the chemotaxis of immune cells (5). After IS, an insufficient blood supply deprives brain cells of glucose and oxygen and disrupts the homeostasis of cells, thereby triggering pathophysiological processes, including excitotoxicity, oxidative stress, inflammation, apoptosis and cell death (1). The immune microenvironment plays an important role in the pathophysiological progression of stroke (6). Blockage of cerebral blood flow produces brain tissue ischemia, which quickly causes a series of chain reactions. Signaling molecules, such as brain-derived antigens, damage-associated molecular patterns (DAMPs), cytokines and chemokines, are released from damaged brain tissue into the systemic circulation. Peripheral immune cells, including macrophages, migrate through the injured blood-brain barrier (BBB) to the site of injury and activate host immune cells, such as microglia (7). Infiltrating macrophages and activated microglia release more cytokines, chemokines, and other molecules, leading to further damage or protection of the ischemic brain (8). T cells play an important role in the inflammatory response in stroke. CD4+, CD8+ and gamma delta (γδ) T cells damage infarcted brain tissue by releasing proinflammatory cytokines (such as IFN-γ and IL-17) (9). In contrast, regulatory T cells (Treg cells) protect brain tissue by releasing anti-inflammatory cytokines (such as IL-10 and TGF-β) (10). Kristian et al. showed that weeks after the onset of IS, activated B lymphocytes enter the brain tissue to mediate chronic inflammation and delay cognitive dysfunction of the nervous system through morphological changes and secretion of antibodies (11). It was found that microglia/macrophages with an LPS-induced M1 phenotype mainly release cytotoxic substances that induce inflammation and lead to cell death, which manifests as a destructive effect on the brain. In contrast, the cells with an IL-4-induced M2 phenotype remove cellular debris and release trophic factors through phagocytosis, which manifests as a neuroprotective effect on the brain (12). Pradillo et al. used an IL-1 receptor antagonist (IL-1RA) in a middle cerebral artery occlusion (MCAO) model, which reduced infarct size by 50% on MRI images (13). The application of an anti-CD49d antibody can inhibit leukocyte infiltration and reduce infarct volume (14). ERK5 is a newly discovered member of the MAPK family. Studies have found that the activation of ERK5 is beneficial for ischemia protection (15). Decreasing JNK1/2 and p38 MAPK activity can reduce the expression of TNF-α and IL-1β and further alleviate the degree of cerebral edema and neurological damage in patients with IS (16). However, the immune microenvironment and inflammatory pathway activation in the peripheral blood of IS patients remain unclear. Traditional experimental methods cannot systematically clarify the functional role or analyze the mechanisms of various factors in the immune microenvironment during the occurrence and development of IS.

Therefore, based on the high-throughput sequencing data of IS samples from the First Affiliated Hospital of Gannan Medical University and the First Affiliated Hospital, Jinan University, and the microarray data of an IS dataset from the Gene Expression Omnibus (GEO) database, we used bioinformatic analysis to compare the differences in the immune microenvironment in the peripheral blood between the IS and control groups. The differences in the degree of activation in the immune microenvironment and inflammatory signaling pathways provide a theoretical basis for the treatment and prevention of IS.

## Methods

### Clinical Samples

**Figure 1:**
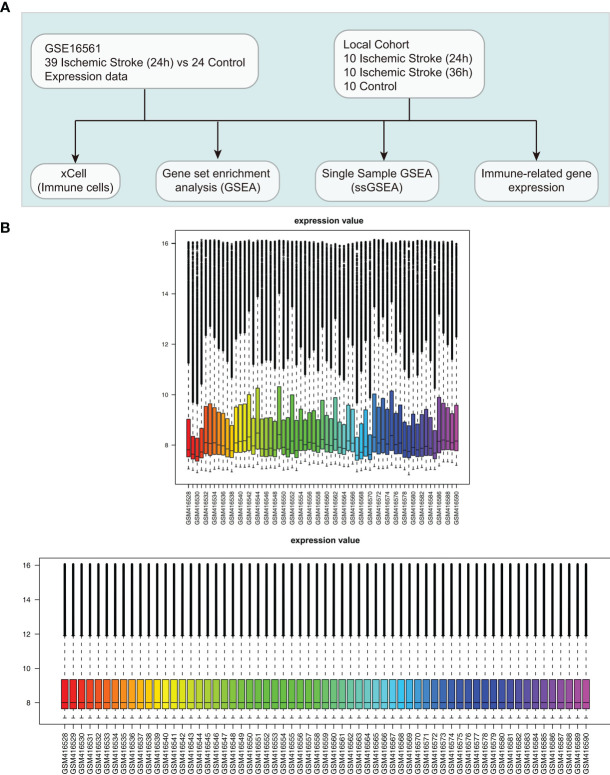
Clinical cohort consolidation. **(A)** Study flow chart. **(B)** Normalization of the expression data in GSE16561.

In the NCBI GEO database (https://www.ncbi.nlm.nih.gov/geo/) (17), we collected one dataset related to IS (ID: GSE16561) for analysis using the “GEOquery” R package (18). The GSE16561 dataset based on the GPL6883 platform includes 39 IS patients and 24 controls (19). We used normalizeBetweenArrays of the limma (20) R package to normalize the expression data in GSE16561. Also, we collected data from the First Affiliated Hospital of Gannan Medical University and The First Affiliated Hospital, Jinan University. Peripheral blood samples (24 h and 36 h) were collected from 10 patients diagnosed with IS at university hospitals and from 10 normal people, which was called Local-IS. Written informed consent was provided by all the patients of Local-IS included in the study. Regarding the diagnostic criteria for IS, the inclusion and exclusion criteria have been reported in the Barr et al. (19). For a detailed description of the RNA-seq analysis of the samples, please refer to the **Supplementary Methods**. **Figure 1A** shows our study workflow. The clinical characteristics of the GSE16561 was detailed in the **Supplementary Table 1**.

### Immune Microenvironment and Inflammatory Signaling Pathway Analysis

The xCell algorithm was used to calculated the scores for immune cell infiltration in the peripheral blood of each patient in the GSE16561 and Local-IS datasets (20). The microenvironmental score and immune score were calculated by the xCell algorithm. This method integrated the advantages of gene enrichment analysis through deconvolution and could evaluate the enrichment of immune cells. Additionally, we collected immune-related genes from the published literature. These immune-related genes (**Supplementary Table 2**) were used to assess inflammation, antigen processing and presentation, cytotoxicity and other functional activities in peripheral blood samples (21, 22). The expression levels of these genes were quantified as log2(FPKM+1). Gene set enrichment analysis (GSEA) was performed using the clusterProfiler R package (23), in which P<0.05 for Gene Ontology (GO) terms, Kyoto Encyclopedia of Genes and Genomes (KEGG) results and Reactome pathways was considered to indicate significant differences. The gene set in this study was from the Molecular Signatures Database (MSigDB) (24). Additionally, the single-sample GSEA (ssGSEA) algorithm was used for the calculation of pathway activity scores for each sample based on gene expression levels (25).

### Flow Cytometry

PBMCs were washed twice and resuspended in RPMI medium (Sigma-Aldrich, USA) supplemented with 5% fetal bovine serum (Gibco, Australia), surface-stained with a PerCP/Cyanine5.5-labeled anti-CD3 antibody, FITC-conjugated anti-CD4, Brilliant Violet 711-labeled anti-CD8, PE-labeled anti-CD45RO and APC-labeled anti-CD62L for 30 min on ice in a dark room. For intracellular analysis of transcription factors, fixation and permeabilization of cells were performed using True-NuclearTM Transcription Factor Buffer Set (Biolegend, USA) according to the manufacturer’s instructions. After washing, the cells were stained with a PE Cyanine7-labeled anti-T-bet antibody in the dark at room temperature for at least 30 min. Fluorescence minus one control tube was also prepared for each antibody. Then, cell sample acquisition and analysis were carried out on a FACSCanto flow cytometer using BD FACSDiva software (BD Biosciences). All antibodies were purchased from Biolegend.

### Quantitative Real-Time PCR

Total RNA was isolated using Trizol reagent (Invitrogen, USA), following the manufacturer’ s instructions. The quantity and purity of the total RNA was measured using with the Nanodrop^®^ ND1000 (Thermo Fisher) and the Agilent Bioanalyzer. The total RNA was reverse transcribed into cDNA using the Script cDNA synthesis kit (AG11728) according to the manufacturer’ s protocol. Quantitative PCR reaction was run on a CFX96 Touch Real-Time PCR Detection Instrument (BioRad, USA), using SYBR^®^ Green Premix Pro Taq HS qPCR Kit(ROX Plus)(AG11718). Primer pairs are shown in **Supplementary Table 3**. Values were normalized to GAPDH *via* the 2^-ΔΔCt^ method.

### Statistical Analysis

For GSE16561, the Mann-Whitney U test was used to evaluate differences in continuous variables between the two groups. For the Local-IS dataset, the Mann-Whitney U test was used to evaluate differences in continuous variables between the IS-24h and control groups or the IS-36h and control groups; the paired Mann-Whitney U test was used to compare continuous variables between the IS-36h and IS-24h groups (paired samples). P < 0.05 was considered statistically significant, and all statistical tests were two-sided tests. All statistical tests and visual analyses were performed with R software (version 3.6.1).

## Results

### Differences in Immune Cells in the Peripheral Blood Between the IS and Control Groups

**Figure 2:**
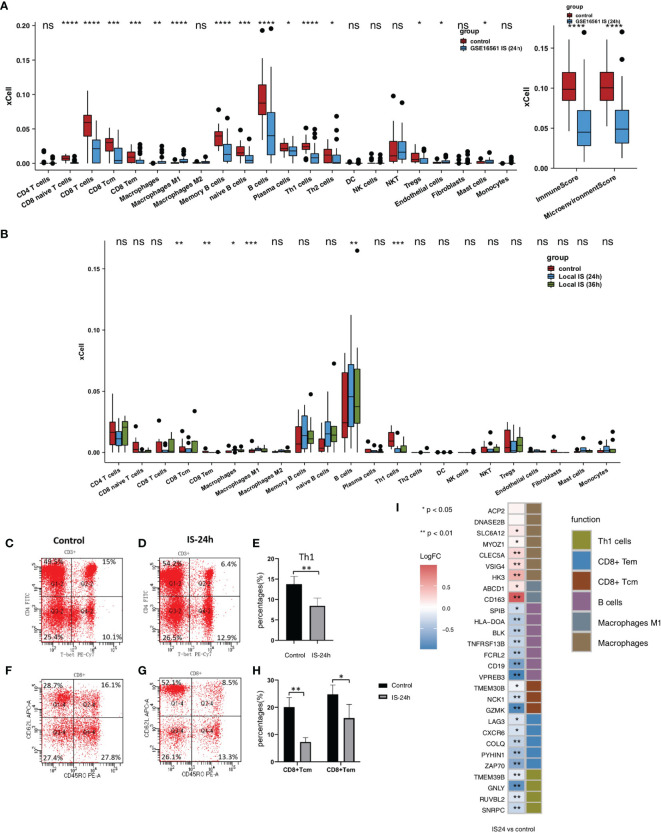
Comparison of the immune cells and cell markers between IS and control group. **(A)** Comparison of the scores for immune cells estimated by the xCell algorithm between the IS-24h and control groups in GSE16561. **(B)** Comparison of the scores for immune cells estimated by the xCell algorithm between the IS-36h, IS-24h and control groups in Local-IS. **(C)** FACS analysis of Th1 cells in the control group of Local-IS. **(D)** FACS analysis of Th1 cells in the IS-24h of Local-IS. **(E)** Percentage of Th1 cells in control and IS-24h. **(F)** FACS analysis of CD8+Tcm and CD8+Tem in the control group of Local-IS. **(G)** FACS analysis of CD8+Tcm and CD8+Tem in the IS-24h of Local-IS. **(H)** Percentage of CD8+Tcm and CD8+Tem in control and IS-24h. **(I)** Comparisons of the cell markers for CD8+ Tcms, CD8+ Tems, B cells, Th1 cells, macrophages and M1 macrophages between the IS-24h and control groups in GSE16561. (*P < 0.05, **P < 0.01, ***P < 0.001, and ****P < 0.0001; Mann-Whitney U test). ns, not significant.

To evaluate the immune infiltration pattern in peripheral blood samples from IS patients, microarray data (GSE16561) were normalized based on the normalizeBetweenArrays method (**Figure 1B**). Next, we used the xCell algorithm to analyze gene expression data (the GSE16561 and Local-IS datasets) to further calculate the immune cell fraction in each peripheral blood sample (**Figure 2**). In GSE16561, the peripheral blood of the IS-24h group had significantly reduced lymphocyte scores, such as those for naive CD8+ T cells, CD8+ T cells, central memory CD8+ T cells (CD8+ Tcms), effector memory CD8+ T cells (CD8+ Tems), memory B cells, naive B cells, B cells, plasma cells, T helper 1 (Th1) cells, Th2 cells, endothelial cells and Treg cells (all P <0.05; **Figure 2A**). In contrast, the scores for M1 macrophages and macrophages in the peripheral blood of IS patients were significantly higher than those of the control participants. Similarly, compared with those in the control group, the peripheral blood samples in the IS-24h group had significantly lower scores and microenvironment scores (all P <0.05, **Figure 2A**). In the Local-IS dataset, the peripheral blood samples in the IS-24h group had lower scores for CD8+ Tcms, CD8+ Tems, B cells and Th1 cells than those in the control group. In contrast, compared with the control group, the IS-24h group had higher macrophage and M1 macrophage scores in the peripheral blood. Similarly, the IS-36h group had significantly lower CD8+ Tcm, CD8+ Tem and Th1 cell scores in the peripheral blood than the control group (all P <0.05; **Figure 2B**). Additionally, a paired Mann-Whitney U test showed that the peripheral blood samples in the IS-36h group had significantly lower CD8+ Tem and M1 macrophage scores than those in the IS-24h group (all P<0.05; **Figure 2B**). Moreover, the peripheral blood of IS patients collected at 36 h was compared with that collected at 24 h, and the fraction of B cells in the peripheral blood was significantly increased (P <0.05; **Figure 2B**). As shown by flow cytometry, the percentage of Th1 cells (gated as CD4+/T-bet) in the control group was significantly higher than that in IS-24 group (p < 0.01) (**Figures 2C–E**). Additionally, significant differences were observed in the numbers of CD8+ Tcms and CD8+ Tems between the control and IS-24 groups (**Figures 2F–H**). Then, we continued to analyze the cell markers CD8+ Tcms, CD8+ Tems, B cells, Th1 cells, macrophages and M1 macrophages [reported by Aran et al. (20)]. For CD8+ Tcm markers, the gene expression levels of GZMK, NCK1 and TMEM30B in the IS-24h group were significantly lower than those in the control group. For CD8+ Tem markers, the LAG3, CXCR6, COLQ, PYHIN1 and ZAP70 levels were significantly lower in the IS-24h group than in the control group. For Th1 cell markers, the expression levels of TMEM39B, GNLY, RUVBL2 and SNRPC in the IS-24h group were significantly lower than those in the control group. In contrast, for the cell markers of macrophages and M1 macrophages, the IS-24h group had significantly increased expression levels of CD163, ABCD1, HK3, VSIG4, CLEC5A, MYOZ and SLC5A12 (**Figure 2I**).

**Figure 3:**
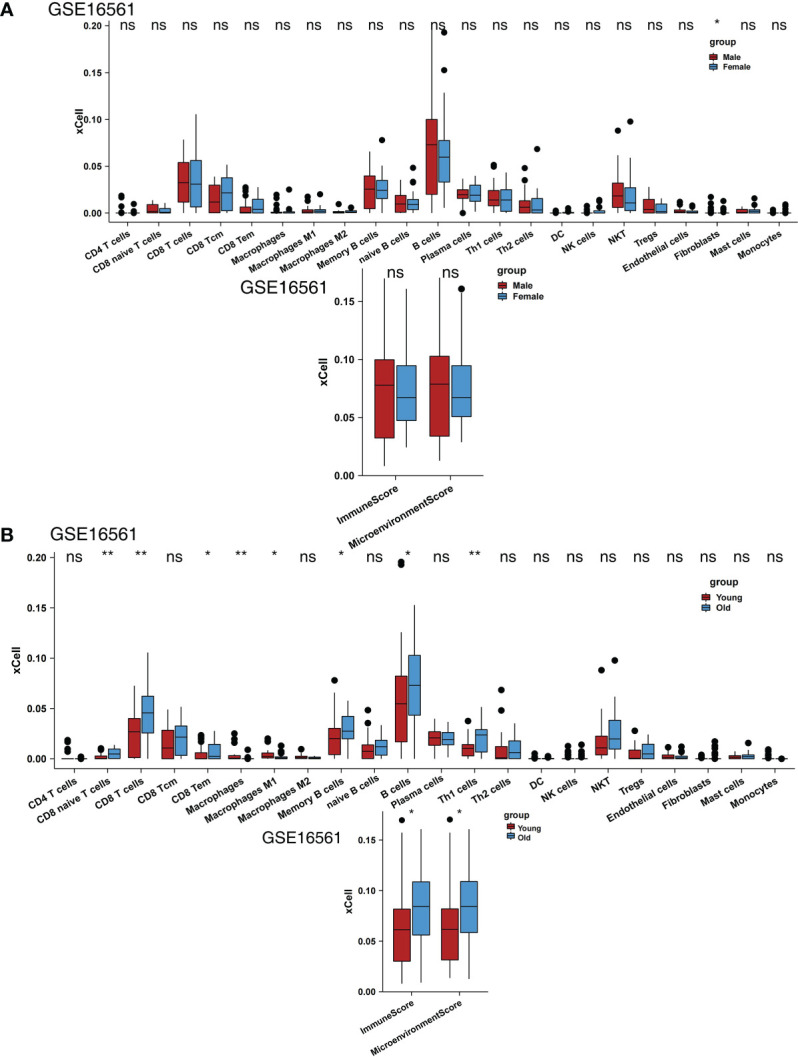
Comparison of the immune cells between clinical subgroups. **(A)** Comparison of the scores for immune cells estimated by the xCell algorithm between males and females in GSE16561. **(B)** Comparison of the scores for immune cells estimated by the xCell algorithm between young and old individuals in GSE16561. (*P < 0.05, **P < 0.01; Mann-Whitney U test). ns, not significant.

We also analyzed the impact of sex on scores, and the results showed that men and women had no significant differences in most scores (**Figure 3A**). We found that older people had significantly higher naive CD8+ T cell, CD8+ T cell, CD8+ Tem, memory B cell, B cell, and Th1 cell scores and immune and microenvironment scores (**Figure 3B**). In contrast, younger people had significantly higher macrophage and M1 macrophage scores (**Figure 3B**). In the GSE16561 and Local-IS datasets, a heatmap of the correlations between differential scores is shown in **Supplementary Figure 1**.

### Pathway Enrichment and Differences in Pathway Scores in the Peripheral Blood Between the IS and Control Groups

**Figure 4:**
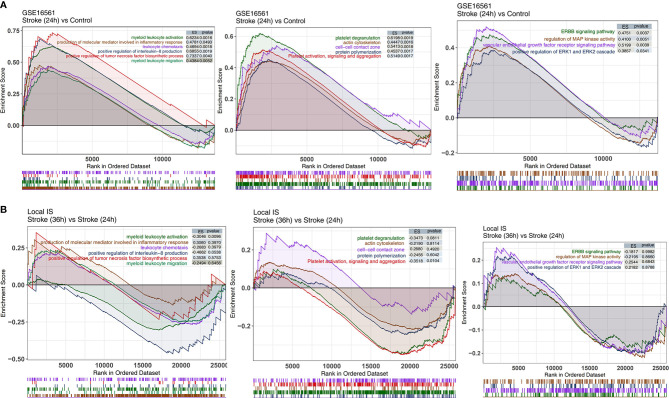
The results of GSEA. **(A)** GSEA results for inflammation-related, platelet-related, and pathological pathways in GSE16561 compared between the IS-24h and control groups. **(B)** GSEA results for inflammation-related, platelet-related, and pathological pathways in the Local-IS cohort compared between the IS-36h and IS-24h groups. All transcripts were ranked by the log2(fold change) between the IS-24h and control groups or IS-36h and IS-24h groups. Each run was performed with 1,000 permutations.

We analyzed whether the IS and control groups had significant differences in the degree of enrichment of some specific pathways in the peripheral blood. We found that there were significant differences in the activities of inflammation-related pathways, pathological pathways, and platelet-related pathways in the peripheral blood between the IS and control groups. In GSE16561, for the inflammation-related pathway, the terms myeloid leukocyte activation, positive regulation of tumor necrosis factor biosynthetic process, positive regulation of interleukin-8 production, myeloid leukocyte migration, leukocyte chemotaxis, and production of molecular mediator involved in inflammatory response were significantly enriched in the IS group (**Figure 4A**). Additionally, the activity of pathology-related pathways, such as the ERBB signaling pathway, positive regulation of ERK1 and ERK2 cascade, vascular endothelial growth factor receptor signaling pathway, and regulation of MAP kinase activity, was significantly higher in the IS group than in the control group (**Figure 4A**). Compared with the peripheral blood samples in the control group, those in the IS group showed enriched terms related to the activities of platelet-related pathways, including platelet activation, signaling and aggregation, protein polymerization, platelet degranulation, cell-cell contact zone, and actin cytoskeleton (**Figure 4A**). Additionally, we also analyzed the differences in pathway enrichment in the peripheral blood between the IS-36h and IS-24h groups and found that the differences in the peripheral blood pathway enrichment between the IS-36h and IS-24h groups might be different from those between the IS-24h and control groups. The activity of myeloid leukocyte activation in the peripheral blood was significantly lower in the IS-36h group than in the IS-24h group (**Figure 4B**). Similarly, platelet activation, signaling and aggregation pathway activities were significantly downregulated in the IS-24h group compared with the IS-36h group (**Figure 4B**). There were 374 DEGs in the IS-36h group compared to the IS-24h group, of which 174 were upregulated and 200 were downregulated (P < 0.05, |logFC|>1; **Supplementary Figure 2A**). The 20 most upregulated and 20 downregulated genes in the IS-36h group compared with the IS-24h group, the IS-36h group compared with the control group and the IS-24h group compared with the control group were visualized in a heatmap (**Supplementary Figures 2B** **–** **D**). Based on the significantly different pathways identified by GSEA of **Figure 4**, the differences in the expression of core genes in the corresponding pathways in the IS group also suggested that there were significant differences in these pathways between the IS and control groups (**Supplementary Figures 3A** **,** **B**).

**Figure 5:**
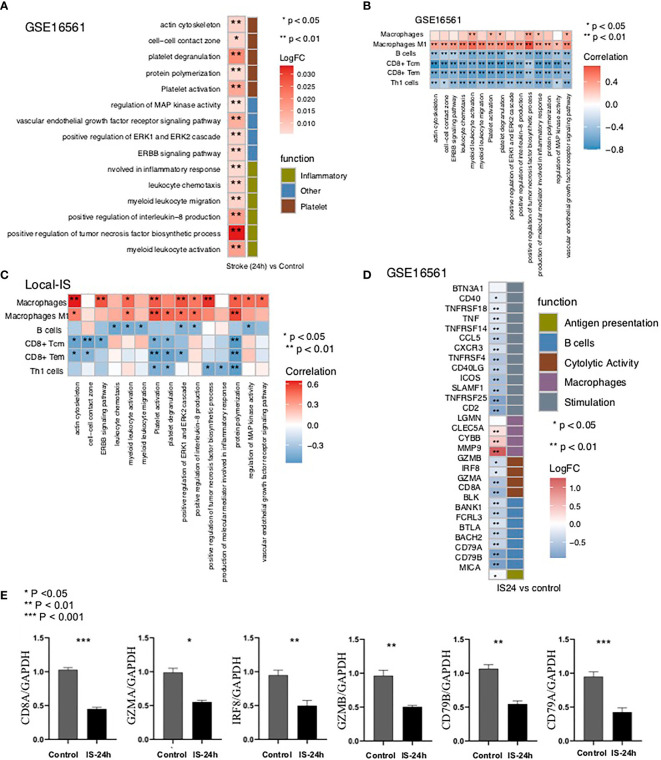
The result of ssGSEA. **(A)** Heatmap depicting the mean differences in ssGSEA scores for several signaling pathways between the IS-24h and control groups in GSE16561. The y axis indicates the inflammation-related, platelet-related, and pathological pathways. Each square represents the fold change or difference in the ssGSEA score for an inflammation-related, platelet-related, or pathological pathway between the IS-24h and control groups. Red indicates upregulation, while blue indicates downregulation. **(B)** Correlation analysis of the scores of different immune cells estimated by the xCell algorithm and the ssGSEA scores for signaling pathways in GSE16561. **(C)** Correlation analysis of the scores for different immune cells estimated by the xCell algorithm and the ssGSEA scores for signaling pathways in the Local-IS cohort. **(D)** Heatmap depicting the mean differences in the mRNA expression of immune-related genes between the IS-24h and control groups in GSE16561. The y axis indicates the immune signature or gene compared between the IS-24h and control groups in GSE16561. Each square represents the fold change or difference in each indicated immune signature or gene between the IS-24h and control groups. Red indicates upregulation, while blue indicates downregulation. **(E)** Expression profile of CD8A, GZMA, IRF8, GZMB, CD79B and CD79A between IS-24h and control groups in Local-IS (qPCR). *P < 0.05; **P < 0.01; ***P < 0.001.

To further verify the pathway activity of the peripheral blood samples in the IS and control groups, we used the ssGSEA algorithm to score the corresponding pathways for each sample in GSE16561 (IS and control groups). The heatmap shows that for the abovementioned inflammation-related pathways, the ssGSEA scores of platelet-related pathways and pathological pathways were significantly higher in the IS-24h group than in the control group (**Figure 5A**).

### Relationships Between the Pathway Score and Scores

To further verify the relationship between the activities of the above differential pathways and differential scores, we conducted a correlation analysis of the ssGSEA scores of these pathways. The results showed that the macrophage score was significantly positively correlated with the ssGSEA scores for inflammation-related pathways, platelet-related pathways and pathological pathways (R> 0, P <0.05; **Figure 5B**). Similarly, we found that a higher M1 macrophage score was related to a higher ssGSEA score for inflammation-related pathways, platelet-related pathways and pathological pathways (P <0.05; **Figure 5B**). In contrast, there were significant negative correlations between the scores for B cells and the scores for the above differential pathways (R <0; P <0.05; **Figure 5B**). Similarly, the higher the CD8+ Tem, CD8+ Tcm or Th1 cell immune score was, the lower the enrichment of the inflammation-related pathways, platelet-related pathways and pathological pathways related to the ssGSEA score (R <0; P <0.05; **Figure 5B**). Then, we also verified this result with the Local-IS dataset (**Figure 5C**). For the GSE16561 and Local-IS datasets, a heat map of the correlations between the ssGSEA scores of these differential pathways is shown in **Supplementary Figure 4**.

Then, we further explored the differences in immune-related signatures in the peripheral blood between the IS and control groups. The expression of macrophage signature genes (MMP9, CYBB and CLEC5A) in the IS group was significantly higher than that in the control group. Additionally, for antigen presentation-related signatures (MICB), cytotoxicity-related signatures (CD8A, GZMA, IRF8 and GZMB), B cell-related signatures (CD79B, CD79A, BACH2, BTLA, FCRL3, BANK1 and BLK) and inflammation-related signatures (CD2, TNFRSF25, SLAMF1, ICOS, CD40LG, TNFRSF4, CXCR3, CCL5, TNFRSF14, TNF, TNFRSF18 and CD40), the expression levels in the IS group were significantly lower than those in the control group (**Figure 5D**). Moreover, the findings regarding the immune-related signatures were validated by the qPCR results (**Figure 5E**). In the qPCR, the expression of CD8A, GZMA, IRF8, GZMB, CD79B and CD79A in the Local IS-24h was significantly lower than that in the control group (**Figure 5E**).

## Discussion

In this study, we aimed to define the immune microenvironment and specifically activated inflammation-related pathways and pathological pathways in the peripheral blood of IS patients. We found that at 24 h, the peripheral blood of IS patients had significantly reduced CD8+ Tcm, CD8+ Tem, B cell and Th1 cell scores and significantly increased macrophage and M1 macrophage scores. Compared with the control group, the IS group had significantly higher inflammation-related pathway activity (myeloid leukocyte activation, positive regulation of tumor necrosis factor biosynthetic process, positive regulation of interleukin-8 production, myeloid leukocyte migration, leukocyte chemotaxis, and production of molecular mediator involved in inflammatory response), platelet-related pathway activity (platelet activation, signaling and aggregation, protein polymerization, platelet degranulation, cell-cell contact zone, and actin cytoskeleton) and pathological pathway activity (ERBB signaling pathway, positive regulation of ERK1 and ERK2 cascade, vascular endothelial growth factor receptor signaling pathway, and regulation of MAP kinase activity). Additionally, immune-related signature analysis showed that the macrophage signature, antigen presentation-related signature, cytotoxicity-related signature, B cell-related signature and inflammation-related signature were significantly lower in the IS group than in the control group.

Inflammation plays an important role in the pathophysiology of stroke. The increase in the permeability of the BBB and the destruction of BBB integrity open the door to invasion and infiltration by peripheral T cells and mediate T cell development in IS, playing an important role in the process (6). Brain infiltration by T cells has been shown to be a key event that promotes inflammatory tissue damage after stroke (26). T cells play a major immune role in IS. In animal models of IS, the cerebral infarct area was reduced in T cell gene knockout mice, while the infarct area increased when the T cell gene was administered. Relevant studies have shown that T cells have an adverse effect on stroke by promoting the adhesion of white blood cells to the cerebrovascular system and triggering thrombotic inflammation in animals (27). CD8+ T cells are cytotoxic T cells (Tcs) that kill target cells. When the number of Tc cells is too high, these cells can cause damage to the body. T cells flow into the brain within a few hours after middle cerebral artery occlusion, and most of them gather at the edge of the infarct area and are located near the blood vessels. Approximately 40% of the T cells infiltrating ischemic brain tissue are CD4+ Th cells, and approximately 30% are CD8+ Tcs. It has been confirmed that CD8+ Tcs can be recruited into the brain within 3 h after stroke, while CD4+ T cells can be recruited within 24 h, with this population reaching a peak at 72 h after reperfusion and then gradually declining (28). CD8+ T cells release perforin/granzyme after antigen activation through cell-cell interactions or cause neuronal death through the Fas-FasL pathway, which has a direct cytotoxic effect on injured cells after cerebral ischemia (29, 30). Wang et al. found that the levels of T cells and natural killer (NK) cells in the peripheral blood of stroke patients were significantly lower than those in the peripheral blood that of normal people and that the levels of T cells and B cells were negatively correlated with the severity of stroke (31). Selvaraj et al. found that CD8+ T cells, CD4+ T cells and NKT cells were recruited within 24 h after ischemic attack and accumulated in the early peak period of inflammation 3~4 d after injury (32). In this study, we found that the IS-24h group had significantly lower CD8+ Tcm, CD8+ Tem and Th1 cell scores than the control group. Additionally, the immune scores of CD8+ Tcm, CD8+ Tem and Th1 cells were significantly lower in the IS-36h group than in the control group. Numerous studies have shown that stroke can induce suppression of peripheral immune function (33). Peripheral immune remodeling 24 h after the onset of IS suppresses the functional state of the immune system, as evidenced by a rapid and sustained decrease in cellular immune function, inactivation of monocytes and Th cells, reduction of lymphocytes, and atrophy of secondary lymphoid organs, a phenomenon known as stroke-induced immunodepression (SIID) (34, 35). Therefore, in this study, the IS-24h group had significantly lower CD8+ Tcms, CD8+ Tems, B cells, and Th1 cells than the control group, consistent with previous findings (33–35). Gill et al. reported that cytotoxic CD8+ T cells were recruited into the brain as early as 3 h following stroke, while CD4+ T cells were recruited within 24 h and peaked at 72 h after reperfusion (36). Additionally, the GSEA and ssGSEA results suggested that myeloid leukocyte activation, positive regulation of IL-8 production, myeloid leukocyte migration, leukocyte chemotaxis, and production of molecular mediators involved in the inflammatory response in the peripheral blood were increased. In light of the important role of T cells in the development of IS, studies have shown that recombinant T cell receptor ligands (RTLs), which are major histocompatibility class II molecules covalently bound to myelin antigens, can stimulate T cell receptors; additionally, RTLs can cause self-reactive T cells to lose their pathogenicity and exert immunomodulatory effects. In cerebral ischemia mouse models, RTLs can reduce cerebral infarction volume and immune inflammation but have little suppressive effect on peripheral immune function (37).

In addition to T cells, B cells and macrophages play important roles in the development of IS (6). Studies have shown that B cells can affect the long-term prognosis of stroke patients. They accumulate in the infarct area 4 to 7 weeks after stroke and produce IgA and IgG antibodies related to cognitive impairment, leading to vascular dementia and sequelae of IS (38). Additionally, studies have shown that B cells are beneficial lymphocytes in the immune response after cerebral ischemia. There is sufficient evidence that B cells are the main immunoregulatory cells in the inflammatory process after ischemic brain injury. B cells can regulate the activation and recruitment of immune cells, such as T cells, macrophages and microglia, after stroke. B cells limit infarct volume and neurological deficits, playing a neuroprotective role in the process of inflammation (39, 40). Regulatory B cells, a subtype of B cells, can inhibit the expansion of proinflammatory T cells, enhance the expansion of Treg cells and secrete anti-inflammatory cytokines (such as IL-10) to suppress the immune response, thereby protecting the brain (41). Studies in mouse models have found that when B cell genes are eliminated, the outcome of stroke can be exacerbated, while adoptive transfer of B cells can limit brain inflammation, reduce mortality, and reduce neurological deficits, indicating that B cells play a neuroprotective role after stroke (42). Proinflammatory factors (such as TNF-α, IL-1β, and IL-6) enable a large number of circulating inflammatory cells (mainly monocytes, macrophages, lymphocytes, etc.) in the peripheral blood to reach the ischemic brain area through the damaged BBB. Within a few hours, the local brain tissue shows hyperactive immune inflammation (43). In our study, the IS-24h group had significantly lower B cell scores for the peripheral blood than the control group. Additionally, the IS group had significantly increased positive regulation of tumor necrosis factor biosynthetic process pathway activity. Maintaining the number of B cells is another way to treat stroke. Sterling et al. (44) transplanted B cells into mice and found that the infarct volume of the mice was reduced at 3 d and 7 d. McCulloch et al. found that β-adrenergic receptor antagonists could prevent the loss of spleen B cells, thereby maintaining the number of spleen B cells and a normal level of immunoglobulin and reducing infections (45). Macrophages are involved in the subacute phase of stroke, and they mainly accumulate in the ischemic area. The infarct area peaks at 72~96 h after ischemia (46). In this study, we found that the IS-24h group had significantly higher scores for macrophages and M1 macrophages in the peripheral blood than the control group. Additionally, the IS-36h group had a significantly reduced M1 macrophage score compared with the IS-24h group. Studies have shown that M2-like macrophages have neuroprotective effects; in contrast, M1-like macrophages have neuroinflammatory effects (47, 48). Studies have found that interferon regulatory factor (IRF), which mediates the activation of macrophages in peripheral immune cells and other inflammatory diseases, can regulate the transition between activation states in macrophages. IRF5 signaling can induce M1 macrophages, while IRF4 signaling induces M2 macrophages. The IRF5-IRF4 regulatory axis can induce mutual M1-M2 conversion to control the proinflammatory and anti-inflammatory responses of macrophages at the genetic level to meet the needs of inflammatory conditions at different stages of stroke (49). Jing et al. found that NOSH-NBP is a hybrid of NO and hydrogen sulfide (H2S) that can promote the conversion of macrophages from the M1 phenotype to the M2 phenotype, greatly reduce the volume of cerebral infarctions, and contribute to nerve function restoration (50). Studies have indicated that macrophages may be an effective target for the treatment of IS. Other studies suggest that facilitating the M2 phenotype and suppressing the M1 phenotype are beneficial to brain recovery after stroke (12, 29).

There is growing evidence that inflammatory cells and the immune system play an important role in stroke, and exploring targeted interventions based on immune inflammatory IS therapy and autoantibody markers for risk assessment of cerebral infarction may provide new pathways in the management of acute IS (51–55). Studies have shown that fingolimod reduces the number of CD4+ T cells and CD8+ T cells in peripheral blood and brain tissue of hypoxic-ischemic mice (51). In a phase II clinical trial study, fingolimod reduced brain infarct volume and BBB permeability, as well as the number of CD4+ T cells and CD8+ T cells in the blood of IS patients (52). A phase II clinical trial of the combination of fingolimod and alteplase showed that the two drugs were better tolerated and reduced IS injury (53). PR-957, an inhibitor of the immunoproteasome subunit low-molecular-weight polypeptide 7 (LMP7), provides neuroprotection by inhibiting T cell infiltration and decreasing cytodifferentiation of T helper 17 (Th17) cells in IS mice (54). Recombinant T-cell receptor ligand 1000 (RTL1000) reduces the number of activated monocytes/microglia (CD11b+CD45hi) and CD3+ T cells as well as the percentage of total T cells and neutrophils in the ischemic zone of the mouse brain (55).

IL-1 receptor antagonists (IL-1Ras) are competitive antagonists of pro-inflammatory cytokines and have been widely used in the treatment of inflammatory diseases such as rheumatoid arthritis. Studies have confirmed that IL-1Ra can reduce the volume of cerebral infarction in a rat model of IS. Phase II clinical trials have shown that IL-1Ras can reduce plasma levels of IL-6 and plasma C-reactive protein in patients with IS, and IL-1Ras are well tolerated and have a good safety profile (56). Currently, the specific mechanism regarding the thrombolytic effect of IL-1Ras is unclear. Citalopram is a selective 5-hydroxytryptamine reuptake inhibitor, clinically used as an antidepressant. Experimental studies have shown that citalopram downregulates the expression levels of IL-1β, TNF-α and adrenocorticotropin-releasing hormone in the hypothalamus after IS in rats (57). However, in a phase II clinical study, it was found that treatment with citalopram during the first 6 months did not improve the symptoms of IS patients (58). Most of the studies on citalopram in IS in recent years have focused on the antiplatelet, neuroregenerative and vascular protective functions, while studies on the anticytokine functions are rare.

Although this study analyzed the immune microenvironment of the peripheral blood of patients with IS and tried to clarify the possible mechanism involving the immune microenvironment in the occurrence and development of IS, there were still some limitations. First, this study included only two IS cohorts, and the assessment of the immune microenvironment in IS peripheral blood might be biased. Second, this study did not develop animal models to elucidate the role of the immune microenvironment in the pathogenesis of IS. Finally, we will include more time points in the future.

## Conclusions

In this study, we found that peripheral blood from IS patients and controls had different immune microenvironments, as shown by the IS group having significantly reduced scores for CD8+ Tcms, CD8+ Tems, B cells and Th1 cells and significantly increased scores for macrophages and M1 macrophages. Additionally, inflammation-related, pathological, and platelet-related pathway activities were significantly higher in the IS group than in the control group. We hope that these findings will provide theoretical guidance for the prevention of IS and treatment of IS patients.

## Data Availability Statement

The datasets presented in this study can be found in online repositories. The names of the repository/repositories and accession number(s) can be found in the article/**Supplementary Material**.

## Ethics Statement

Ethical review and approval was not required for the study on human participants in accordance with the local legislation and institutional requirements. Written informed consent for participation was not required for this study in accordance with the national legislation and the institutional requirements.

## Author Contributions

Conceptualization, YZ and ZQ. Formal analysis, RL, PS, XG, and WL. Visualization, RL, PS, XG, and WL. Writing—original draft, WS and QH. Writing—review and editing, YZ, ZQ, RL, PS, XG, WL, WS, and QH. All authors contributed to the article and approved the submitted version.

## Funding

This work was supported by the Science and Technology Project of Education Department of Jiangxi Province (180805), the Science and Technology Project of Health and Family PlanningCommission of Jiangxi Province (20195363), the CSCO-Haosen Research Fund (Y-HS2019/2-015), the Fundamental Research Funds for the Central Universities (21620406), the Natural Science Foundation of Guangdong Province (2020A1515011249) and the Science and Technology Project of Ganzhou City (GZ2021SF002).

## Conflict of Interest

The authors declare that the research was conducted in the absence of any commercial or financial relationships that could be construed as a potential conflict of interest.

## Publisher’s Note

All claims expressed in this article are solely those of the authors and do not necessarily represent those of their affiliated organizations, or those of the publisher, the editors and the reviewers. Any product that may be evaluated in this article, or claim that may be made by its manufacturer, is not guaranteed or endorsed by the publisher.

## Supplementary Material

Supplementary Figure 1 Correlation analysis of the scores for immune cells estimated by the xCell algorithm in the GSE16561 **(A)** and Local-IS cohorts **(B)**.

Supplementary Figure 2A Volcano plot of the AD-control, red represents up-regulated genes, blue represents down-regulated genes, and black represents no significantly differentially expressed genes.

Supplementary Figure 2B A heatmap of 20 most up-regulated and 20 most down-regulated genes in the IS-36h group compared with IS-24h group (Local-IS).

Supplementary Figure 2C A heatmap of 20 most up-regulated and 20 most down-regulated genes in the IS-36h group compared with control group (Local-IS).

Supplementary Figure 2D A heatmap of 20 most up-regulated and 20 most down-regulated genes in the IS-24h group compared with control group (Local-IS).

Supplementary Figure 3A Heatmap of core genes in enriched pathways (the same as those in **Figure 4**) between IS-24h and control group (GSE16561).

Supplementary Figure 3B Heatmap of core genes in enriched pathways (the same as those in **Figure 4**) between IS-36h and IS-24h group (Local-IS).

Supplementary Figure 4 Correlation analysis of the ssGSEA scores for several signaling pathways in the GSE16561 **(A)** and Local-IS cohorts **(B)**.

Supplementary Table 1 The clinical characteristics of the Local-IS.

Supplementary Table 2 The gene list of the immune genes related to **Figure 5D**.

Supplementary Table 3 Primers for qPCR.
