# Supplementary figures and images for: Comprehensive Landscape of Immune Infiltration and Aberrant Pathway Activation in Ischemic Stroke
Source: Front Immunol. 2022 Jan 24;12:766724. doi: 10.3389/fimmu.2021.766724 (PMC8818702; doi:10.3389/fimmu.2021.766724)

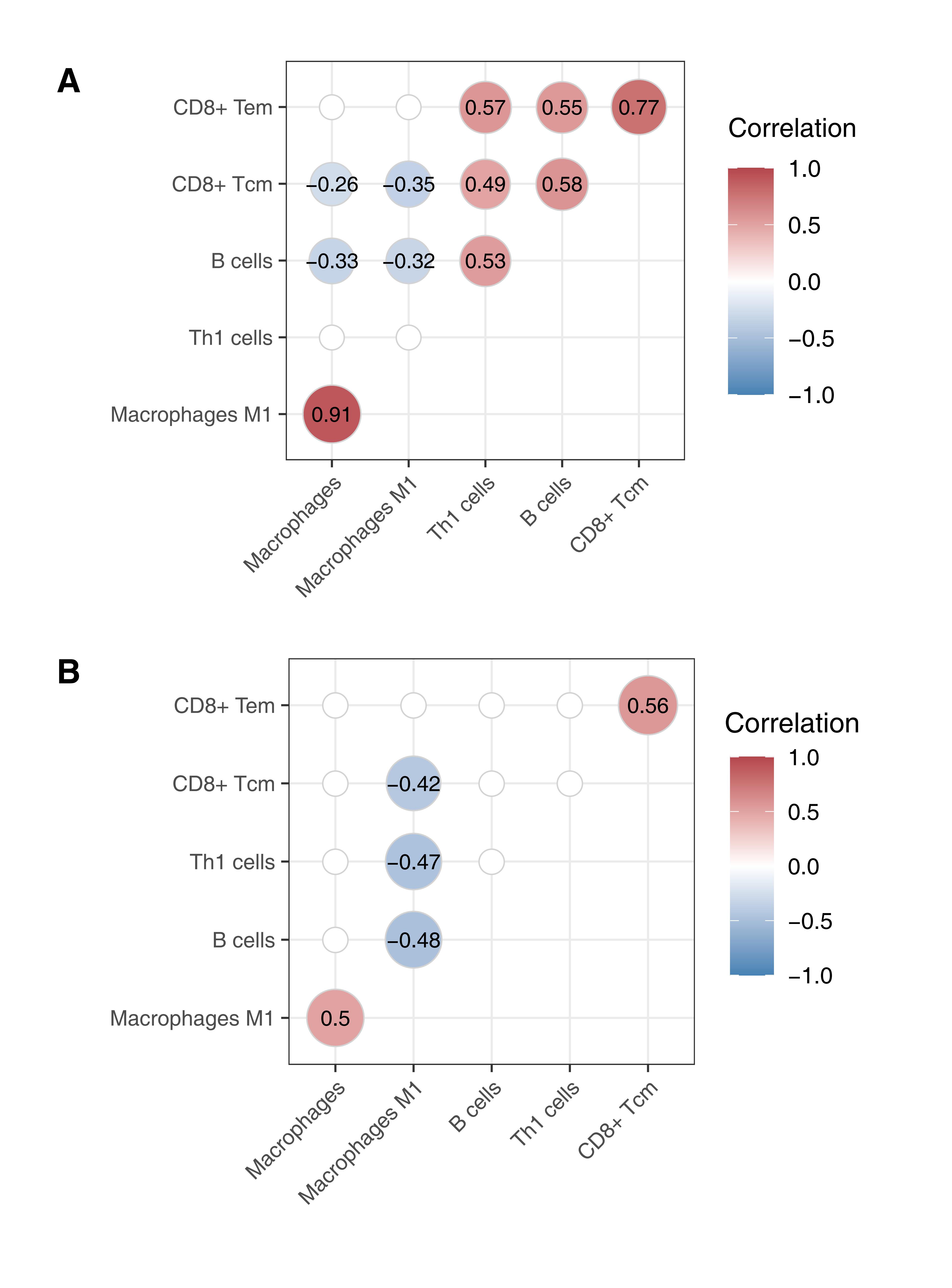

Supplement: Supplementary Figure 1 — Correlation analysis of the scores for immune cells estimated by the xCell algorithm in the GSE16561 (A) and Local-IS cohorts (B). [file Image_1.jpeg]

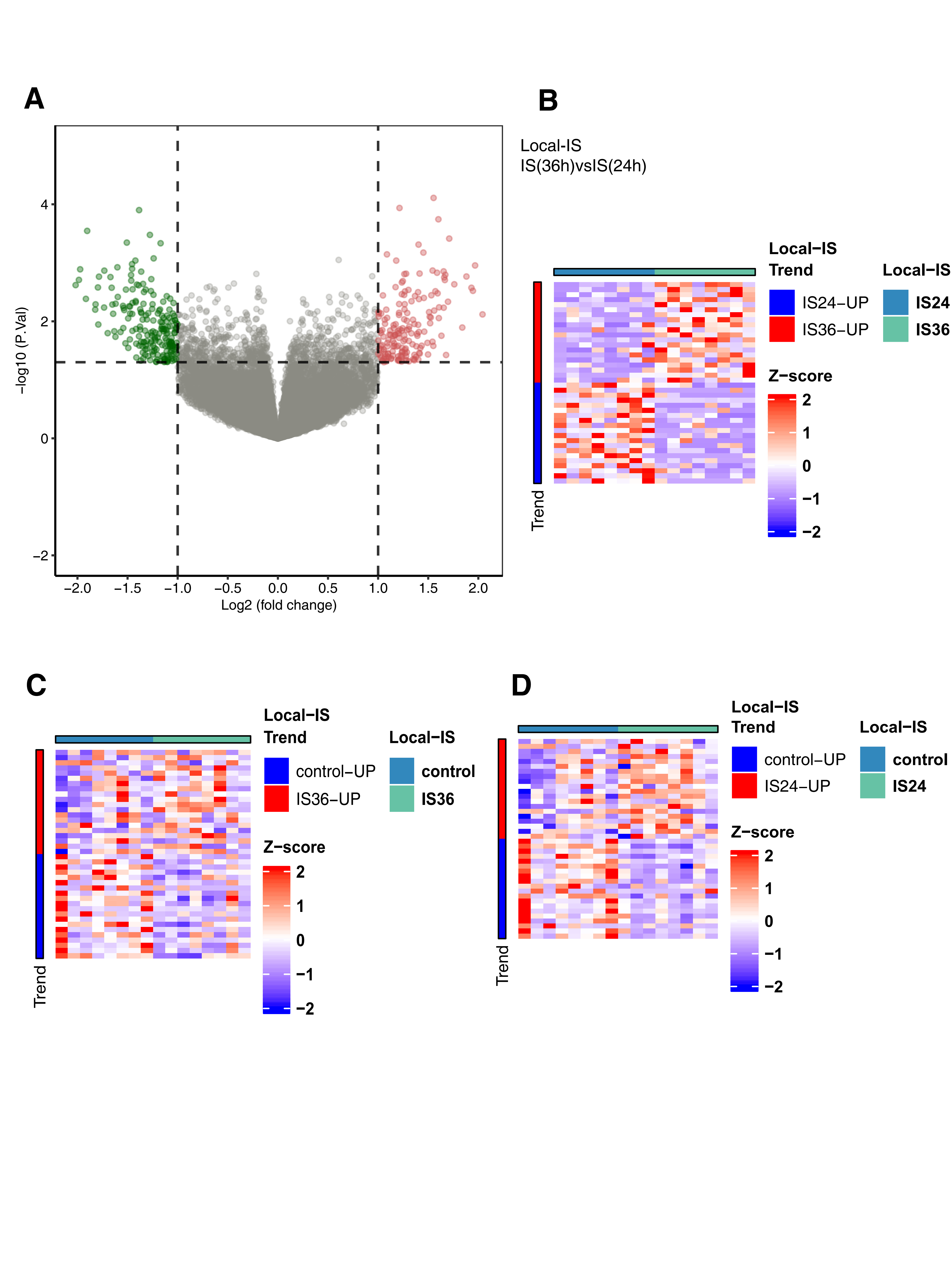

Supplement: Supplementary Figure 2A — Volcano plot of the AD-control, red represents up-regulated genes, blue represents down-regulated genes, and black represents no significantly differentially expressed genes. [file Image_2.png]

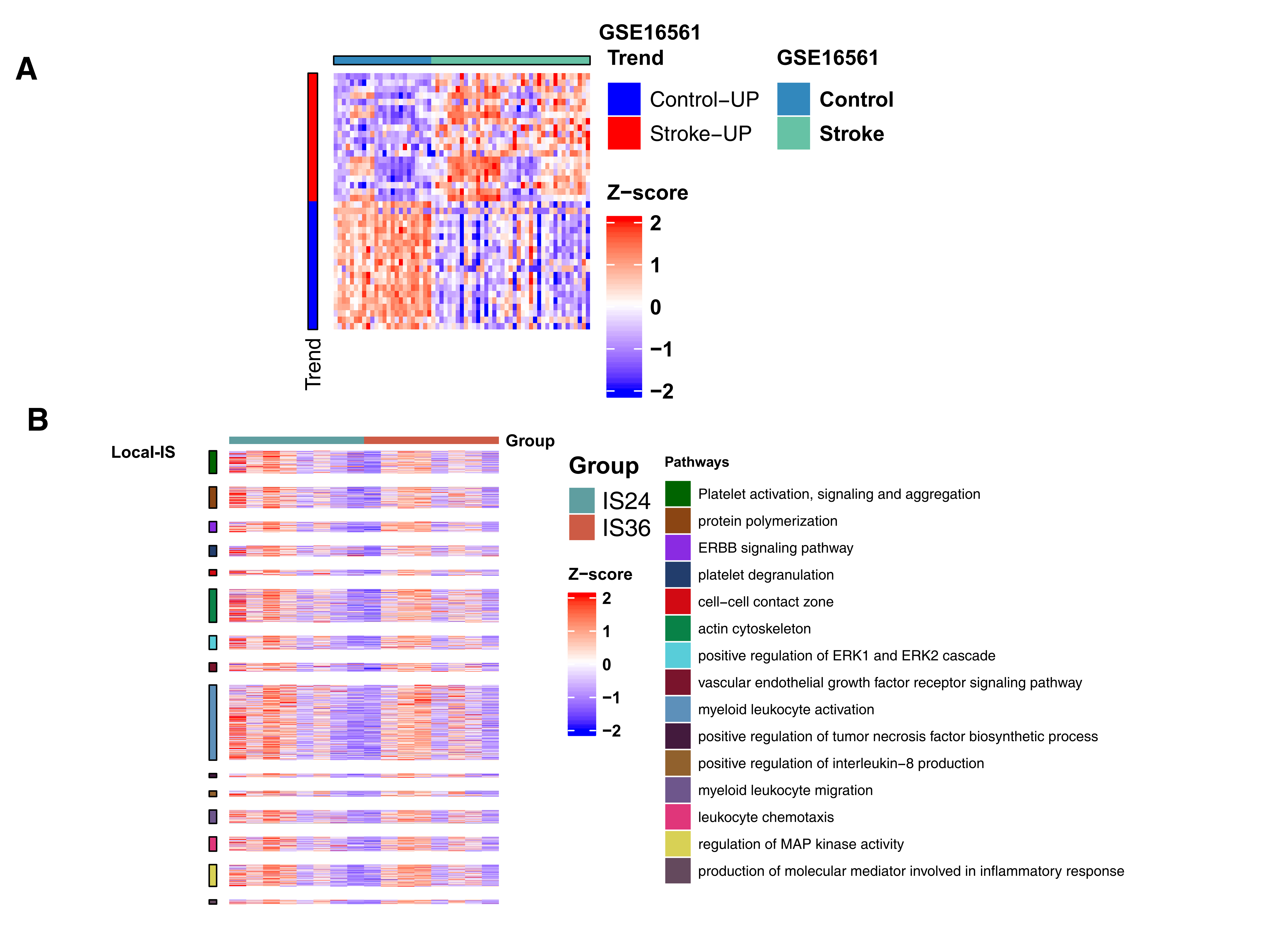

Supplement: Supplementary Figure 3A — Heatmap of core genes in enriched pathways (the same as those in Figure 4 ) between IS-24h and control group (GSE16561). [file Image_3.png]

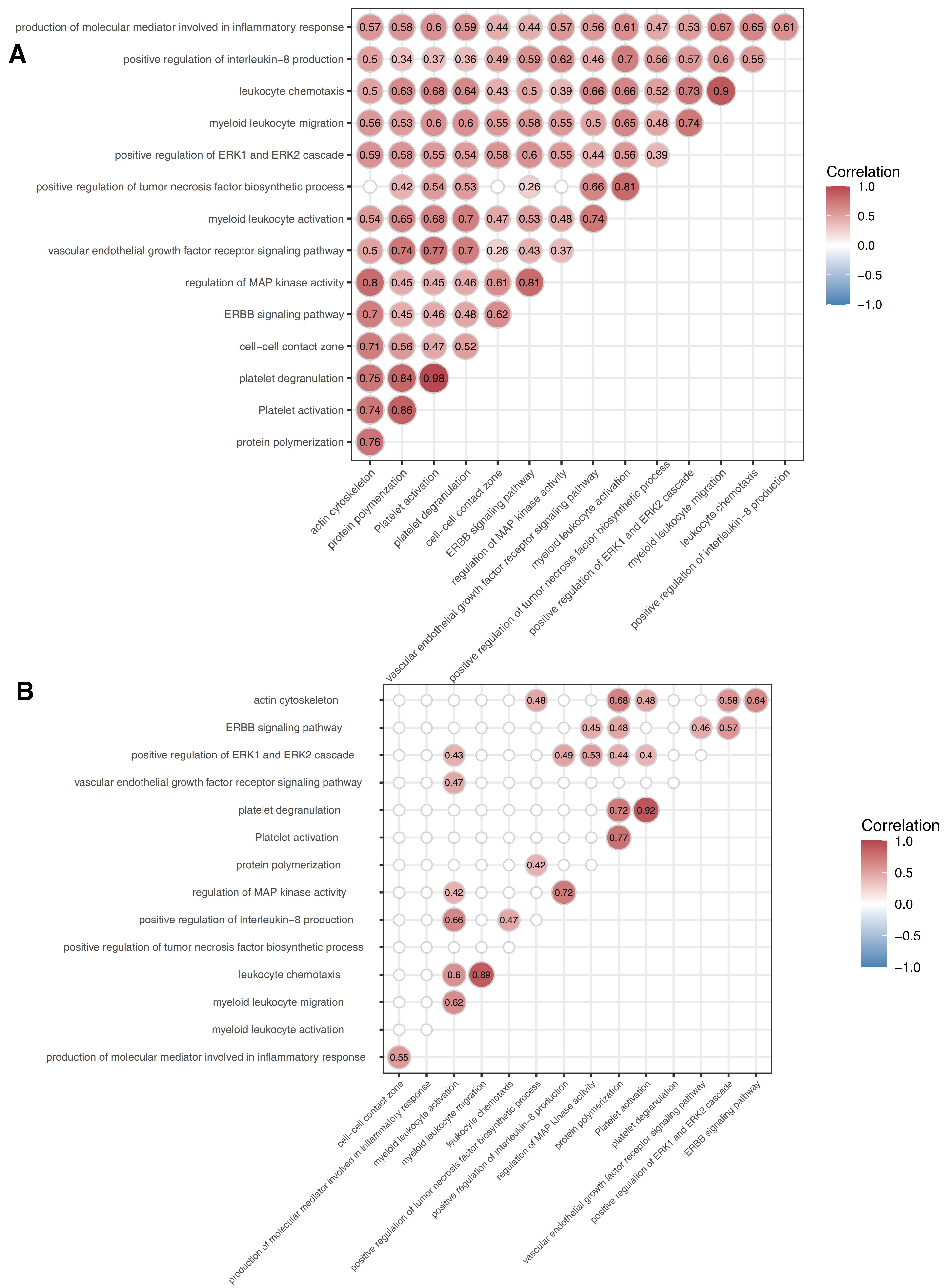

Supplement: Supplementary Figure 4 — Correlation analysis of the ssGSEA scores for several signaling pathways in the GSE16561 (A) and Local-IS cohorts (B). [file Image_4.jpeg]
