# Supplementary material for: Comprehensive Landscape of Immune Infiltration and Aberrant Pathway Activation in Ischemic Stroke
Source: Front Immunol. 2022 Jan 24;12:766724. doi: 10.3389/fimmu.2021.766724 (PMC8818702; doi:10.3389/fimmu.2021.766724)
Supplement: Supplementary Table 1 — The clinical characteristics of the Local-IS. [file Table_1.pdf]

	Control (N=24)	Stroke (N=39)	Overall (N=63)
**Gender**			
Female	14 (58.3%)	22 (56.4%)	36 (57.1%)
Male	10 (41.7%)	17 (43.6%)	27 (42.9%)
**Age**			
Mean (SD)	59.9 (9.73)	73.1 (14.0)	68.0 (14.0)
Median [Min, Max]	59.5 [40.0, 82.0]	77.0 [43.0, 96.0]	68.0 [40.0, 96.0]
