# Supplementary material for: Comprehensive Landscape of Immune Infiltration and Aberrant Pathway Activation in Ischemic Stroke
Source: Front Immunol. 2022 Jan 24;12:766724. doi: 10.3389/fimmu.2021.766724 (PMC8818702; doi:10.3389/fimmu.2021.766724)
Supplement: Supplementary Table 2 — The gene list of the immune genes related to Figure 5D . [file Table_2.pdf]

Symbol	Function	Reference
MICA	Antigen presentation	doi: 10.1016/j.immuni.2018.03.023.
CD79B	B cells	doi: 10.1016/j.cell.2014.12.033.
CD79A	B cells	doi: 10.1016/j.cell.2014.12.033.
BACH2	B cells	doi: 10.1016/j.cell.2014.12.033.
BTLA	B cells	doi: 10.1016/j.cell.2014.12.033.
FCRL3	B cells	doi: 10.1016/j.cell.2014.12.033.
BANK1	B cells	doi: 10.1016/j.cell.2014.12.033.
BLK	B cells	doi: 10.1016/j.cell.2014.12.033.
CD8A	CD8+ T cells	doi: 10.1016/j.cell.2014.12.033.
GZMA	Cytolytic Activity	doi: 10.1016/j.cell.2014.12.033.
GZMB	Cytolytic Activity	doi: 10.1016/j.cell.2014.12.033.
MMP9	Macrophages	doi: 10.1016/j.cell.2014.12.033.
CYBB	Macrophages	doi: 10.1016/j.cell.2014.12.033.
CLEC5A	Macrophages	doi: 10.1016/j.cell.2014.12.033.
LGMN	Macrophages	doi: 10.1016/j.cell.2014.12.033.
CXCR3	pDCs	doi: 10.1016/j.cell.2014.12.033.
IRF8	pDCs	doi: 10.1016/j.cell.2014.12.033.
CD2	Stimulation	doi: 10.1016/j.cell.2014.12.033.
TNFRSF2[illegible]	Stimulation	doi: 10.1016/j.cell.2014.12.033.
SLAMF1	Stimulation	doi: 10.1016/j.cell.2014.12.033.
ICOS	Stimulation	doi: 10.1016/j.cell.2014.12.033.
CD40LG	Stimulation	doi: 10.1016/j.cell.2014.12.033.
TNFRSF4	Stimulation	doi: 10.1016/j.cell.2014.12.033.
CCL5	Stimulation	doi: 10.1016/j.immuni.2018.03.023.
TNFRSF1[illegible]	Stimulation	doi: 10.1016/j.immuni.2018.03.023.
TNF	Stimulation	doi: 10.1016/j.immuni.2018.03.023.
TNFRSF1[illegible]	Stimulation	doi: 10.1016/j.cell.2014.12.033.
CD40	Stimulation	doi: 10.1016/j.cell.2014.12.033.
BTN3A1	Stimulation	doi: 10.1016/j.immuni.2018.03.023.
