# Supplementary material for: Comprehensive Landscape of Immune Infiltration and Aberrant Pathway Activation in Ischemic Stroke
Source: Front Immunol. 2022 Jan 24;12:766724. doi: 10.3389/fimmu.2021.766724 (PMC8818702; doi:10.3389/fimmu.2021.766724)
Supplement: Supplementary Table 3 — Primers for qPCR. [file Table_3.pdf]

Gene	Primers(forward)	Primers(reverse)
CD8A	5′-CACCCATGCCATCTCAACCTCTTC-3′	5′-GCTCCAACCCTGACTTGCTGTG-3′
GZMA	5′-AGGTTGATTGATGTGGGACAGCAG-3′	5′-GCAGGAGAGAAACGACAACTGAGAG-3′
IRF8	5′-ACTGCTGGCTGCGTGAATGAAG-3′	5′-GTAATCGTCCACAGAAGGCTCCTTG-3′
GZMB	5′-AGGTGCGGTGGCTTCCTGATAC-3′	5′-CTGGGTCGGCTCCTGTTCTTTG-3′
CD79B	5′-GGGATTCAGCACCTTGGCACAG-3′	5′-CAGCAGGAAGATAGGCACGATGATG-3′
CD79A	5′-TCTTCCTCCTCTTCCTGCTGTCTG-3′	5′-CGTTGGCGTTGTTGCTGCTATTG-3′
GAPDH	5′-GGTCGGAGTCAACGGATTTGGTCG-3′	5′-CCTCCGACGCCTGCTTCACCAC-3′
