# Supplementary material for: Comprehensive Landscape of Immune Infiltration and Aberrant Pathway Activation in Ischemic Stroke
Source: Front Immunol. 2022 Jan 24;12:766724. doi: 10.3389/fimmu.2021.766724 (PMC8818702; doi:10.3389/fimmu.2021.766724)
Supplement: Supplementary file 8 [file DataSheet_1.docx]

**Supplementary Methods**

**Samples and procedures**

Baseline peripheral whole blood samples were collected from stroke patients (primary and validation study) and control participants into Paxgene blood RNA tubes (PreAnalytiX, Qiagen). The tubes were stored in a -80 °C freezer. All patients had a blood draw performed at baseline (24 h) and follow-up (36 h). Control participants had one blood draw performed at baseline only. The study protocol to obtain peripheral blood mononuclear cells (PBMCs) from patients and controls was approved by the Ethical Committee of the First Affiliated Hospital of Gannan Medical University and The First Affiliated Hospital, Jinan University. Before being enrolled, the subjects participating in the study signed an informed consent form.

**Isolation of human PBMCs and RNA-seq library preparation, sequencing and analysis**

PBMCs were prepared by centrifugation. Peripheral blood was layered (density = 1.077) and centrifuged at 950 g for 30 min. After isolation from the Ficoll-Histopaque interface (Sigma-Aldrich, Italy), cell viability was assayed by a trypan blue exclusion test, and cells were then used for RNA extraction. Subsequent RNA extractions were performed with the Qiagen RNeasy Mini Kit according to the instructions provided by the manufacturer. RNA quality was assessed with a Bioanalyzer 2100 DNA Chip 7500 (Agilent Technologies), and samples with an RNA integrity number (RIN) greater than 7 were further analyzed by RNA-seq. All sequencing reactions were performed on an Illumina HiSeq 2000 instrument (Illumina, San Diego, CA, USA). cDNA libraries were prepared from poly(A)-selected RNA by applying the Illumina TruSeq protocol for mRNA. The libraries were then sequenced with a 2- x 100-bp paired-end protocol. We used HISAT2 (version 2.1.0)(1) with the default setting to map the RNA-seq data to a human reference genome (NCBI38/hg38). We aggregated the read counts at the gene level using HTSeq(2).

**Isolation of Peripheral Blood Mononuclear Cells (PBMCs)**

Fresh blood samples (5 mL) were drawn from ischemic stroke patients within 24 h of onset and collected into ethylenediamine tetraacetic acid (EDTA) tubes (BD, USA). Samples were also collected from healthy donors. PBMCs were isolated by density-gradient centrifugation (Histopaque-1077®; Sigma Aldrich, USA).

1. Kim D, Langmead B, Salzberg SL. HISAT: a fast spliced aligner with low memory requirements. *Nat Methods* (2015) **12**:357–360. doi:10.1038/nmeth.3317

2. Anders S, Pyl PT, Huber W. HTSeq--a Python framework to work with high-throughput sequencing data. *Bioinformatics* (2015) **31**:166–169. doi:10.1093/bioinformatics/btu638
